# Sclerotic bone: a sign of bone reaction in patients with medication related osteonecrosis of the jaw

**DOI:** 10.1038/s41598-024-57635-5

**Published:** 2024-04-04

**Authors:** Katharina Theresa Obermeier, Ina Dewenter, Yoana Malenova, Riham Fliefel, Gabriele Kaeppler, Sven Otto

**Affiliations:** 1 Department of Oral and Maxillofacial Surgery and Facial Plastic Surgery, Ludwig-Maximilians-University (LMU), Lindwurmstrasse 2a, 80337 Munich, Germany; 2 Department of Orthopaedics and Trauma Surgery, Musculoskeletal University Center Munich (MUM), Ludwig-Maximilians-University (LMU), Fraunhoferstrasse 20, 82152 Planegg/Martinsried, Germany; 3 Department of Oral and Maxillofacial Surgery, Alexandria University, Alexandria, Egypt

**Keywords:** Bisphosphonate-Associated Osteonecrosis of the Jaw, Antiresorptive drugs, Bone mineral density, Bone regeneration, Infection, Inflammation

## Abstract

Medication-related osteonecrosis of the jaw (MRONJ) is a serious adverse reaction associated with antiresorptive drugs such as bisphosphonates and denosumab. When dealing with advanced and/or multiple MRONJ lesions undergoing surgical therapy, the extent of surgery is often a topic of discussion. The aim of this study was to identify the differences in bone density in and around the MRONJ lesion before and after surgical treatment to evaluate the needed surgical extend of the modelling osteotomy. In this retrospective study 26 patients with MRONJ lesions that were surgically treated in our department were observed. Length, width and bone density were measured in panoramic radiograph pre and postoperatively with the Imaging processing software Sidexis and ImageJ (Fiji). The necrotic area, the surrounding sclerotic area as well as the healthy contralateral side were observed. Measurements were performed by two independent observers. Pearson correlation was calculated to determine the interobserver variability. Bone density was significantly reduced in the necrotic bone area compared to the healthy unaffected contralateral reference side. The sclerotic bone area surrounding the necrosis showed increased bone density compared to the contralateral unaffected reference side. The density of the sclerotic bone area was increased in the previously affected MRONJ area in the postoperative panoramic radiograph. The pre and postoperative density showed no significant correlation to healing behaviour. The focus of the modelling osteotomy in surgical treatment of mature MRONJ lesions should be predominantly on the parts that appear necrotic and less dense in the panoramic radiograph as sclerotic areas might be an expression of bone reaction.

## Introduction

Medication-related osteonecrosis of the jaw (MRONJ) is a severe adverse reaction caused by antiresorptive therapy (AR) or antiangiogenic therapy (AA)^1^. Typical clinical findings are necrotic alveolar bone, infection of the surrounding tissues and fistulas^2^. Around 1–15% of all patients with AR develop MRONJ in the course of disease^3^. The dose of AR, the frequency of application, hygiene of the oral cavity and co-medications play a role in the pathogenesis of MRONJ^4–6^.

The pathophysiology of MRONJ is still not fully elucidated which makes the treatment of MRONJ a challenge to surgeons^7^. Different therapy approaches have been performed as conservative treatment with antibiotics^8^, surgical treatment including osteotomy and removing of the sequestra and the surrounding soft tissue^9^, and even more radical approaches like mandibular resection followed by reconstruction^10,11^. Imaging is recommended to evaluate the preoperative extent of the necrosis. Panoramic radiography is still the imaging method of choice for a routine dental assessment in these patients^12^ and it is the most available dental imaging technique with a low radiation dose. Typical findings are osteolytic areas corresponding to the necrotic area. In some cases sequestra of the bone can be found^13^ and most of the patients show osseous sclerosis around the necrotic area. Also new bone formation and possible narrowing of the mandible canal are described^14^.

The extent of surgical treatment is often discussed as radical treatment can influence quality of life of patients due to aesthetic limitations, facial dysmorphisms or speech impairment^15^. The sclerotic zone that forms around the necrosis is often thought to be a pathological bone change and is therefore surgically removed^16^. However, considering the physiological bone metabolism, this zone is rather regarded as new formation of bone as a reaction to the inflammation^17^. Therefore the aim of this study was to identify the differences in bone density in panoramic radiograph in and around the MRONJ lesion before and after surgical treatment to evaluate the needed surgical extend of the modelling osteotomy.

## Material and methods

This retrospective study was approved by the institutional review board of the University Hospital of the Ludwig-Maximilians-University Munich, Germany (Munich, Germany; UE Nr 22-0445). Informed consent was waived by the institutional review board of the University Hospital of the Ludwig-Maximilians-University Munich, Germany (Munich, Germany; UE Nr 22-0445) due to the retrospective nature of the data. All research was performed in accordance with the guidelines of the Declaration of Helsinki. It includes results of patients treated in an ambulant setting or as patients in our hospital between 2014 and 2022. Patients´ medical records as demographic data, number of lesions per patient, healed vs non-healed lesion, bisphosphonate medication (sort of bisphosphonate, dosis, duration of intake, underlying disease of intake) as well as pre- and postoperative panoramic radiograph have been screened, evaluated and reported according to the STROBE (STrengthening the Reporting of OBservational studies in Epidemiology) guidelines. All methods were performed in accordance with the guidelines and regulations of this journal.

### Inclusion criteria

Patients with pre- and postoperative panoramic radiograph were included in this study. Patients with MRONJ caused by antiresorptive drugs (bisphosphonates and denosumab) with Stage I, II and III in the maxilla or the mandible were included. For the diagnosis of MRONJ lesions the definition of the American Association of Oral and Maxillofacial Surgeon´s positioning paper (update 2022) was used^18^. Only patients who received surgical therapy were included. All patients received conservative therapy prior to surgical treatment including long-term antibiotic treatment, as well as local disinfectant measures, especially locally disinfectant rinses. Patients with more than one lesion were included, as long as the lesions were not located in the same jaw in order to measure the contralateral healthy side as a reference.

### Exclusion criteria

**Figure 1:**
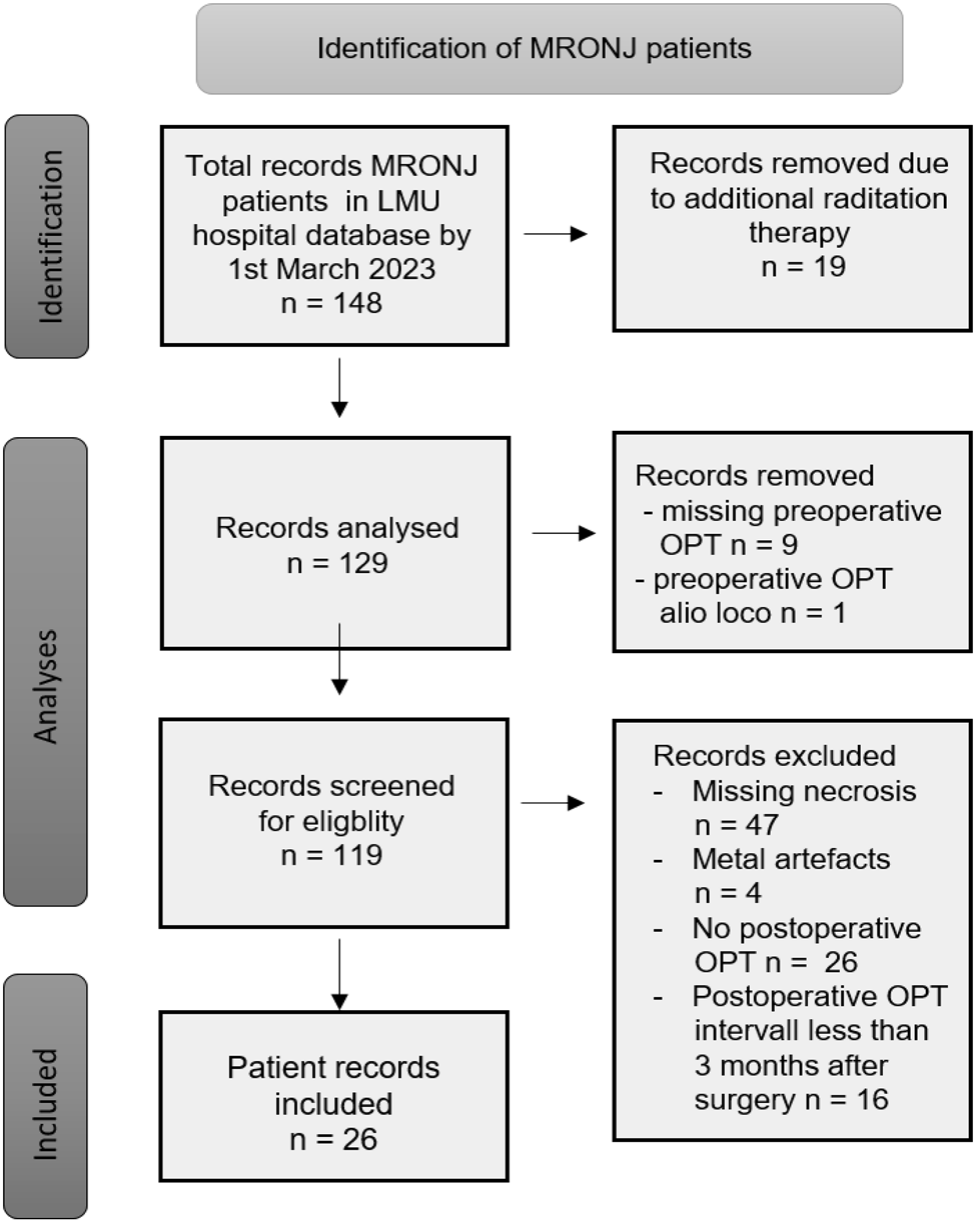
Identication process of MRONJ patients.

Patients with osteoradionecrosis were excluded. All patients with alio loco panoramic radiograph were excluded. Patients with low-quality panoramic radiograph (wrong positioning, too much overlap in the region of interest, metal artefacts) and without clearly visible necrosis in the panoramic radiograph (only clinical exposure of necrotic bone) were excluded. The preoperative panoramic radiograph must not be older than 2 weeks when surgery was performed. Patients with previous lesions were excluded and patients with postoperative panoramic radiograph less than 3 months after surgery were excluded as well. 148 patients were screened for eligibility. 26 patients were included in this study. The identification process of included patients is shown in Fig. 1.

**Figure 2:**
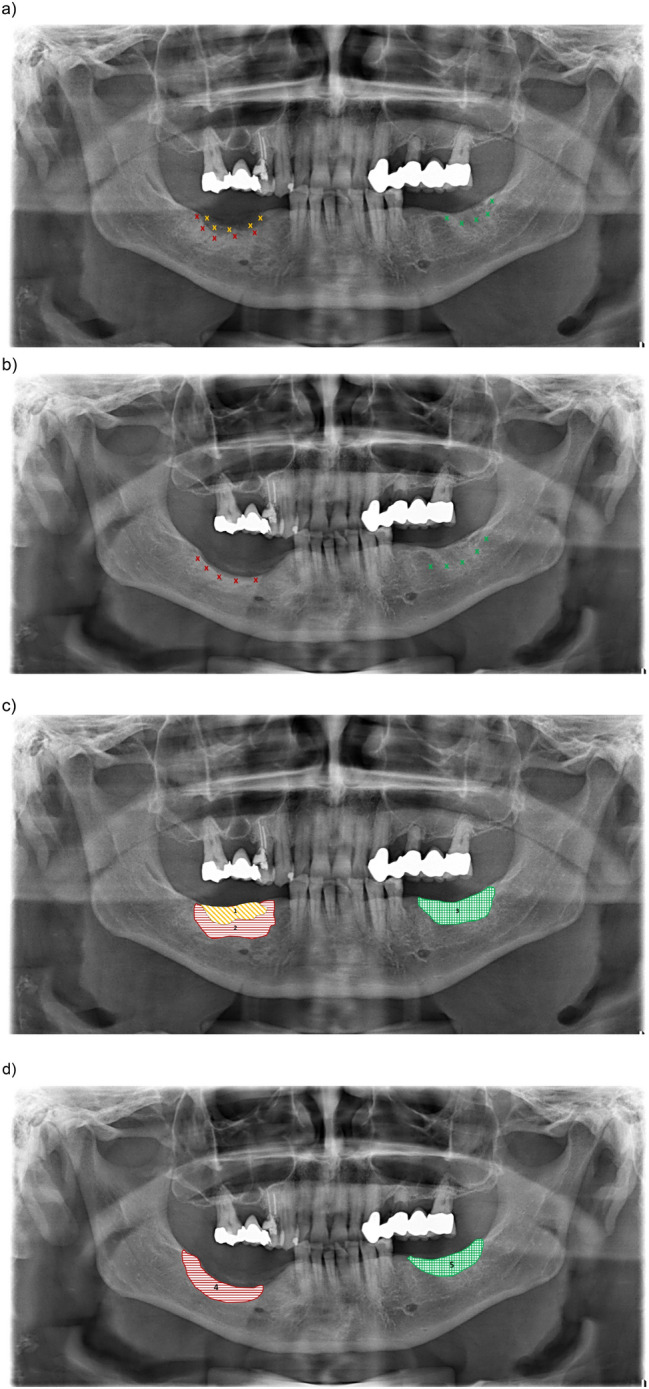
Pre and postoperative panoramic radiograph (**a**,**b**) measuring points (Sidexis), (**c**,**d**) measured bone zones (ImageJ): 1: osteonecrosis; 2: preoperative sclerotic area; 3: preoperative unaffected reference side; 4: postoperative reactive sclerotic area; 5: postoperative unaffected reference side.

All patients received surgical treatment. Surgical treatment of MRONJ included exploration of the affected area and visualization of the entire extent of the lesion. The necrotic bone areas were resected visually and under fluorescence control. This enabled targeted and standardized therapy with reduced invasiveness. Resection was followed by a tight, tension-free wound closure if possible, in multiple layers. Depending on the localization of the defect, fibers of the mylohyoid muscle or the corpus adiposum buccae were prepared as an additional layer as part of the wound closure, mobilized vestibularly over the alveolar ridge and fixed there. Operations were performed under local anesthesia or intubation anesthesia combined with local anesthesia depending on the size of the defect and the patients constitution. In addition pre and postoperative antibiotic therapy (3 g Ampcillin/Sulbactam or 600 mg Clindamycin in case of penicillin allergy three times per day) was applied in all patients. All panoramic radiographs were taken in our department with Orthophos XG (Sirona Dental System) and equal exposition settings to avoid bias. Postoperative panoramic radiographs were performed with a minimum interval of 3 months post-operation. The density of the necrotic zone, the reactive sclerotic zone around the necrosis and the postoperative sclerotic zone were measured with Sidexis 4 (Dentsply Sirona, Charlotte, North Carolina, USA) and are reported in percent. Five points were measured equally distributed in the above localizations respectively. For a healthy reference, five mirror-image points were measured on the opposite unaffected reference side (URS) in each case. In case that the mirrored points overlapped with anatomical structures of a different density, these were slightly displaced so that the reference points were located in healthy bone areas. To further validate the findings, the complete zones were measured again with the image processing programme Fiji (ImageJ, U. S. National Institutes of Health, Bethesda, Maryland, USA) and are reported in numeric grey value. All measurements were performed by two independent observers. Pearson correlation was calculated to determine the interobserver variability. An example of the methods is shown in Fig. 2.

### Statistical analysis

Qualitative and quantitative data were observed, normally distributed data were presented using mean ± standard deviation (SD). Data were tested for normal distribution with the Shapiro–Wilk test. An unpaired t-test was used for the comparison of preoperative necrosis and preoperative URS, preoperative sclerosis and URS and postoperative sclerosis and postoperative URS. For comparisons between pre- and post-operative values especialle sclerosis pre and postoperative a paired t-test was selected for hypothesis testing. The significance level was defined at p ≤ 0.05. Statistical analysis was conducted using SPSS® 24 version 4.0 (SPSS Inc., Chicago, IL, USA).

## Results

26 patients were included in this study. 15 patients (57.7%) were female and 11 (42.3%) male. The mean age at time of first diagnosis of the MRONJ lesion was 70.20 years. Tables 1 and 2 show demographic data, the underlying disease that required AR and the AR itself. Patients with Zolendronate 4 mg or Pamidronate 30 mg received the medications intravenously, patients receiving Denosumab received the medication subcutaneous. Pearson correlation was calculated to determine the interobserver variability. Pearson correlation for the Preoperative URS was 0.6, 0.7 for the necrotic area, 0.6 for the preoperative sclerotic area, 0.7 for the postoperative sclerotic area and 0.6 for the postoperative URS. The average length of the necrotic area measured on panoramic radiograph was 25.3 ± 10.3 mm, the average width was 14.6 ± 7.5 mm. The preoperative density of the necrotic zone was 29.9 ± 5.5% (Sidexis) and 77.6 ± 13.4 (ImageJ). On the URS the medium bone density amounted 42.0 ± 5.0% and 101.1 ± 16.7. The sclerotic area around the necrotic area was 46.9 ± 4.0% and 108.7 ± 15.4. Postoperatively the density of the sclerotic bone area amounted 46.2% ± 4.1% and 112.7 ± 13.4 compared to the URS with 42.8% ± 5.3% and 101.1 ± 16.7. The unpaired t-test showed a statistically significant difference between the density of the necrotic zone and the preoperative URS in both measurement techniques (p = 0.00). The preoperative sclerotic zone around the MRONJ lesion showed a statistical significant difference to the UPS (p = 0.00) with Sidexis and with ImageJ (p = 0.04). The postoperative sclerotic zone showed statistical significant higher density values compared to the postoperative URS (p = 0.00) with Sidexis as well as ImageJ (p = 0.01). Tables 3 and 4 show analysis of bone density and p-values. There was no statistical significant correlation between duration of antiresorptive treatment and bone density. In addition two of the 26 patients did not show healing. In both cases, the lesion was located in the lower jaw. The pre and postoperative density showed no significant correlation to healing behaviour. The hypothesis that non-healed bone showed a higher or lower bone density was not confirmed in this study.

**Table 1:** Demographic data.

Variable	Category	No. of patients (%)
Age, years	70,20	
Sex	Male	11 (42.3%)
	Female	15 (57.7%)
Type or antiresorptive drug	Zolendronate	15 (57.7%)
	Pamidronate	1 (3.8%)
	Denosumab	8 (30.8%)
	Zolendronate and denosumab	1 (3.8%)
	Pamidronate and denosumab	1 (3.8%)
Primary disease	Breast cancer	9 (34.6%)
	Prostate cancer	7 (26.9%)
	Renal cell cancer	2 (7.7%)
	Multiple myeloma	3 (11.5%)
	Osteoporosis	4 (15.4%)
	Colon cancer	1 (3.8%)

**Table 2:** Patient data.

Patient	Gender	Age at diagnosis	Underlying disease of intake	Bisphosphonate	Dosis	Duration of intake (months)	Number of lesions	MRONJ stage (AAOMS)	Localization	Sequester	Healed	Recidive	Postoperative imaging timepoint
1	Female	75	Breast cancer	Zolendronate	4 mg	276	1	I	Regio 34–37	no	no	yes	7 months
2	Male	71	Multiple myeloma	Zolendronate + Denosumab	4 mg	48	1	I	Regio 27	no	yes	no	4 months
3	female	47	Breast cancer	Denosumab	120 mg	84	1	II	Regio 37/38	no	no	yes	7 months
4	Female	55	Colon cancer	Zolendronate	4 mg	23	1	III	Regio 16/17 and 22	no	yes	yes, different location	8 months
5	male	58	Renal cell cancer	Zolendronate	4 mg	71	1	I	Regio 44–47	no	yes	no	18 months
6	Female	68	Breast cancer	Zolendronate	4 mg	42	1	III	Regio 16/17	no	yes	Yes, different location	80 months
7	Male	83	Multiple myeloma	Zolendronat	4 mg	54	1	I	Regio 44–48	no	yes	yes	6 months
8	Female	69	Osteoporosis	Zolendronate	4 mg	48	1	I	Regio 25–27	no	yes	no	12 months
9	Male	66	Prostate cancer	Zolendronate	4 mg	61	1	II	Regio 36–38	no	yes	yes	9 months
10	Female	56	Renal cell cancer	Pamidronat + Denosumab	-	95	1	II	Regio 34–37	no	yes	yes	19 months
11	Female	71	Breast cancer	Zoledronat	4 mg	108	1	II	Regio 45	no	yes	no	5 months
12	Female	62	Breast cancer	Denosumab	120 mg	58	1	III	Regio 22–25	no	yes	Yes, different location	5 months
13	Male	72	Prostate cancer	Zoledronat	4 mg	44	1	I	Regio 12–17	no	yes	no	5 months
14	Female	77	Osteoporosis	Denosumab	60 mg	80	1	II	Regio 47	no	yes	Yes, different location	9 months
15	Female	49	Breast cancer	Denosumab	120 mg	24	1	I	Regio 43	no	yes	yes	3 months
16	Male	72	Prostate cancer	Zolendronate	4 mg	-	1	I	Regio 36	no	yes	no	80 months
17	Female	66	Breast cancer	Pamidronat	30 mg	35	1	I	Regio 15–17	no	yes	no	5 months
18	male	72	Prostate cancer	Zolendronate	4 mg	48	1	III	Regio 35–38	yes	yes	no	6 months
19	male	86	Prostate cancer	Zolendronate	4 mg	138	1	II	Regio 44–48	yes	yes	no	6 months
20	Female	84	Breast cancer	Zolendronate	4 mg	36	1	III	Regio 34–38	no	yes	yes	7 months
21	Male	80	Multiple myeloma	Zolendronate	4 mg	36	2	I	Regio 35–38 and 12–15	no	yes	yes	10 months
22	female	83	Breast cancer	Denosumab	60 mg	60	1	I	Regio 32–34	no	yes	no	15 months
23	Male	72	Prostate cancer	Zolendronate	4 mg	45	1	I	Regio 38	no	yes	Yes, different location	34 months
24	Female	79	Osteoporsoe	Denosumab	60 mg	84	1	II	Regio 47	no	yes	Yes, different location	65 months
25	Female	80	Osteoporosis	Denosumab	60 mg	72	1	I	Regio 33–37	no	yes	Yes, different location	15 months
26	Male	72	Prostate cancer	Denosumab	120 mg	108	1	III	Regio 14–18	no	yes	no	6 months

**Table 3:** Statistical analysis of density measurement (Sidexis).

		p-value
Density preoperative sclerotic zone	Density necrotic zone	0.00
Density preoperative sclerotic zone	Density reference site	0.00
Density necrotic zone	Density reference site	0.00
Density postoperative reactive sclerotic zone	Density postoperative reference site	0.00
Density preoperative sclerotic zone	Density postoperative sclerotic zone	0.81

**Table 4:** Statistical analysis of density measurement (ImageJ).

		p-value
Density preoperative sclerotic zone	Density necrotic	0.00
Density preoperative sclerotic zone	Density reference site	0.04
Density necrotic zone	Density reference site	0.00
Density postoperative sclerotic zone	Density postoperative reference site	0.01
Density preoperative sclerotic zone	Density postoperative sclerotic zone	0.09

## Discussion

Surgery has been described as an effective therapeutic regimen for reducing pain in patients suffering from MRONJ who did not respond to conservative treatment^19^. Surgical treatment includes sequestrectomy, debridement, resection and immediate reconstruction and may also include extraction of teeth within exposed necrotic bone^20^. In the treatment of MRONJ lesions, there is still disagreement about the extent of resection. While some authors suggest a more conservative approach^21^, such as sequestrectomy and surgical debridement others advocate more aggressive therapies, such as resections of affected bone with bigger reconstruction including free flaps and microsurgical reconstruction.

The major challenge in surgical treatment in MRONJ is the delineation between necrotic and viable bone to ensure complete removal of necrotic bone while preserving as much vital bone as possible^22^. Wilde et al. recommend a full-thickness mucoperiosteal flap that should be high and extended to reveal the entire area of exposed bone until disease-free margins are localized; resection of the affected bone should be extended as far as healthy-appearing, bleeding bone is reached considering to smooth sharp edges and primary soft tissue closure is achieved^23^. Seth et al. analysed outcomes of vascularized bone graft reconstruction of the mandible in medication-related osteonecrosis of the jaw; in eleven patients a complication rate of 36% was reported. Complications included infection, fistula, hematoma, pneumonia, deep vein thrombosis and free flap loss^24^. Similar results were reported by Hanasono et al. who report a complication rate of 46%^25^.

Meanwhile it has been shown that a moderate surgical treatment results in better healing since the periosteum and unaffected bone are preconserved^26^. In a case of a 73 year old women with bisphonate therapy for 10 years who was diagnosed with stage III MRONJ lesion, panoramic radiographs revealed a 3-cm-long lesion on the body of the mandible starting from the mental foramen and extending to the posterior region with a reactive bone formation as a result of periosteal activation. Necrotic bone was removed while protecting the periosteum; as a result the 1-year follow-up panoramic radiographs showed complete regeneration of the bone^27^.

The radiographic features of MRONJ remain relatively unspecific. In the early stages of the disease plain radiography does not typically demonstrate abnormalities of the affected bone. Cortical bone thickening and increased trabecular bone density are suspected to be early imaging features of MRONJ^28^. In addition osteolysis, mandibular canal enhancement, and bone sclerosis have already been described as typical findings of different MRONJ stages in panoramic radiograph^29^. Computed tomography diagnostic imaging findings are assumed to be more sensitive to changes in bone mineralization, compared to panoramic radiography^30^. Even though CT often shows a greater quality of bone changes, still, radiographic findings in panoramic radiography such as sclerosis, cortical irregularity, lucency, mottling, fragmentation/sequestra formation, sinus communication, and persistent sockets are reported to have the ability to characterize the extent of the MRONJ lesion as well^31^.

Regarding the imaging modality specific guidelines for routine clinical care have not yet been recommended^32^. While in CT the evaluation of Hounsfield Units (HU) is a valid tool for assessing bone density, in plain radiography, the observation of bone density through gray values in ImageJ is a validated tool in dentistry^33^ as well as in orthopedics^34^. Therefore, it remains to be considered individually whether the increased radiation exposure of a CT is actually relevant for the treatment decision.

Fluorescence-guided bone surgery of medication‐related osteonecrosis of the jaw can actually help to visualise vital bone intraoperatively due to autofluorescence of vital bone^35^. This technique may help to define the transitions between necrotic and none-necrotic bone during the surgical procedure providing a controllable therapeutic approach^36^. Still as this method is not always available other methods to assess the extent of surgical resection are needed.

As shown in our data, density meausrements with Sidexis and ImageJ showed a significant difference in bone density between the preoperative sclerotic appearing zone and the healthy unaffected reference bone (p = 0.00). In addition the necrotic areas showed a significant reduction in bone density compared to the URS. This indicates that the focus of the modelling osteotomy should be predominantly on the parts that appear necrotic and osteolytic in the panoramic radiograph. Sclerotic areas are already described as an expression of bone reaction during initial bone healing phase^37,38^ and should therefore not be removed. This observation can be explained by the principles of bone metabolism: in damaged bone, apoptotic osteocytes signal the location and size of damage to lining cells leading to the formation of the bone remodelling compartment (BRC). The BRC confines and targets remodeling to the damage in order to minimize the removal of normal bone^39^. As bisphosphonates bind strongly at sites of mineral deposition in osteoid as well as to resorption sites, they do not only inhibit osteoclastic bone resorption in physiological remodelling, but also in pathological processes^40,41^, indicating that the usage of antiresorptive agents such as bisphosphonates or denosumab increases bone density^42^ independently on the formation of MRONJ lesions.

This goes along with the finding, that the pre- and postoperative density showed no significant correlation to healing behaviour. The hypothesis that non-healed bone showed a higher bone density was not confirmed in this study and reinforces the hypothesis, that preoperative appearing sclerotic bone should not be removed. Postoperative appearing sclerosis as in our data has also been observed in other bone entities as a result of bone reaction after osteotomy. Even after segmental mandibulectomy in MRONJ patients sclerosis was observed in the postoperative CT^16^. In addition it was also shown in tibial osteotomies that the sclerosis appearing postoperative in the conventional radiograph proved to be new bone in the CT and could therefore be identified as reliable parameter for ossification^43^.

In this study the density of the different bone areas was identified comparing pre-and post-operative panoramic images measured by two independent observers. Pearson correlation showed an acceptable inter rater reliability in choosing the extent of necrotic and sclerotic zones, still, interrater bias are limitations of this method described in this retrospective study, even though this is also faced when segmenting CT or CBCT. In addition positioning of the patient during the radiograph might also influence the image density values. In order to minimize measurement bias, contrast as well as exposition and machine settings were set to fixed standardized parameters when acquiring the image. In Germany it is mandatory to perform a monthly constancy test using test specimen for dental x-ray systems to guarantee correct resolution, contrast and radiation field. This procedure acts as a quality control for equal imaging parameters reducing the risk of imaging bias in our study.

Therefore, ImageJ and Sidexis Density Analysis might offer a reliable tool for clinicians to improve preoperative analysis and planning on the extent of the modelling osteotomy in MRONJ lesions. It offers an option to avoid extensive surgery resulting in large defects of the jaw which further influence postoperative dental rehabilitation and quality of life^44,45^. Methods like ImageJ and Sidexis density analysis are fast and easy available and are therefore easily included in pre- and postoperative imaging assessment in osteonecrosis of the jaw. As the above described methods are even applicable in ambulant dental practice that perform regular dental screening in order to prevent or diagnose MRONJ in an early stage in patients with antiresorptive drugs, this methodology might also detect MRONJ lesions in early stages.

In addition advantage of the here presented methology is the low radiation exposure of the patients. In most cases there is no justifying indication for both, pre and post-operative CBCT. Therefore this method is a low radiation alternative for bone density evaluation in the jaw. Still larger studies analysing both, bone density in panoramic radiography and CT in MRONJ are desirable to further validate our findings.

## Conclusion

Sclerotic-appearing bone areas around the necrosis in MRONJ lesions showed a higher bone density pre- and postoperatively compared to the healthy unaffected side. The pre- and postoperative density showed no significant correlation to healing behaviour. The hypothesis that non-healed bone showed a higher bone density was not confirmed in this study. Sclerotic areas around necrotic bone in MRONJ lesions might be an expression of bone reaction, and should therefore not be removed. Density measurements with Sidexis and ImageJ in the panoramic radiograph could be established as a handable option for dentists and oral and maxillofacial surgeons to evaluate MRONJ lesions without 3D imaging. The extent of the sclerotic area could be used as a surgical guide for the extent of surgery and modelling osteotomy.

## Data Availability

The datasets generated and/or analysed during the current study are not publicly available due but are available from the corresponding author on reasonable request, as during the data evaluation process all data has been anonymised.
